# Nano-sized precipitate stability and its controlling factors in a NiAl-strengthened ferritic alloy

**DOI:** 10.1038/srep16081

**Published:** 2015-11-05

**Authors:** Zhiqian Sun, Gian Song, Jan Ilavsky, Gautam Ghosh, Peter K. Liaw

**Affiliations:** 1 Department of Materials Science and Engineering, The University of Tennessee, Knoxville, TN 37996, USA; 2 X-ray Science Division, Argonne National Laboratory, Lemont, IL 60439, USA; 3 Department of Materials Science and Engineering, Northwestern University, Evanston, IL 60208, USA

## Abstract

Coherent B2-ordered NiAl-type precipitates have been used to reinforce solid-solution body-centered-cubic iron for high-temperature application in fossil-energy power plants. In this study, we investigate the stability of nano-sized precipitates in a NiAl-strengthened ferritic alloy at 700–950 °C using ultra-small angle X-ray scattering and electron microscopies. Here we show that the coarsening kinetics of NiAl-type precipitates is in excellent agreement with the ripening model in multicomponent alloys. We further demonstrate that the interfacial energy between the matrix and NiAl-type precipitates is strongly dependent on differences in the matrix/precipitate compositions. Our results profile the ripening process in multicomponent alloys by illustrating controlling factors of interfacial energy, diffusivities, and element partitioning. The study provides guidelines to design and develop high-temperature alloys with stable microstructures for long-term service.

In order to improve thermal efficiency and reduce greenhouse gas emissions, fossil-energy power plants require an increase of the steam temperature and pressure^1^,^2^,^3^. In the United States, endeavors are underway to push the steam temperature to 760 °C and steam pressure to 35 MPa^3^. Higher steam temperatures and pressures impose new challenges on materials in fossil-energy power plants, for example, the creep resistance decreases. A tolerable creep strain rate under operation conditions in fossil-energy power plants is estimated to be ~3 × 10^−11^ *s*^−1^ [ref.^4^]. Nowadays, the most advanced creep-resistant ferritic steels are 9–12%Cr ferritic steels^5^,^6^ and they can only operate at a metal temperature up to ~620 °C^1^.

Using precipitation hardening^7^,^8^,^9^,^10^, coherent B2-ordered NiAl-type precipitates have been employed to strengthen solid-solution body-centered-cubic (bcc) iron^11^,^12^,^13^,^14^,^15^,^16^,^17^,^18^ for elevated-temperature application in fossil-energy power plants. Considerable previous research has revealed underlying creep mechanisms of NiAl-strengthened ferritic alloys^11^,^15^,^16^. Based on our study, a NiAl-strengthened ferritic alloy, designated as FBB8 (see Table 1 for its compositions), exhibits superior creep rupture strength at 700 °C as compared to commercial ferritic heat-resistant steels, P122 and P12.

However, nano-sized strengthening features (e.g., particles/precipitates and dislocation substructures) are thermally unstable at high temperatures. Specifically, secondary particles/precipitates tend to grow and coarsen driven by decreasing the total interfacial energy. The detailed thermodynamic procedure was given by Ratke and Voorhees^19^. Theoretically, Lifshitz and Slyozov^20^ and Wagner^21^ (LSW) first developed a way to describe the coarsening behavior of a secondary phase in a dilute binary system. Later, research extended the LSW theory to multicomponent systems with a considerable secondary phase^19^,^22^,^23^,^24^,^25^. A general ripening theory in multicomponent alloys was recently developed by Philippe and Voorhees^26^. Microstructural degradation was reported in 9–12%Cr ferritic steels^27^,^28^,^29^ and nickel-based superalloys^30^,^31^,^32^ and it significantly weakens their creep resistance for long-term service, such as in fossil-energy power plants^33^,^34^. Considering the microstructural instability, Ennis *et al.*^35^ suggested that the development of new 9–12%Cr steels for application at 600 °C and above would be challenging. However, few studies have attempted to understand the stability of NiAl-type precipitates in the bcc iron matrix for application in next-generation fossil-energy power plants^36^,^37^,^38^.

In this study, we investigate the coarsening behavior of NiAl-type precipitates in FBB8 at 700–950 °C using combined experimental methods, including the ultra-small angle X-ray scattering (USAXS), scanning electron microscopy (SEM), and transmission electron microscopy (TEM). The coarsening kinetics is examined by the ripening model in multicomponent alloys^26^ and its controlling factors of interfacial energy, diffusivities, and alloying element partitioning are discussed. The study provides a systematic view concerning phase stability in materials, and helps design and develop advanced alloys with stable microstructures for high-temperature application.

## Results

### Characterization by SEM

**Figure 1:**
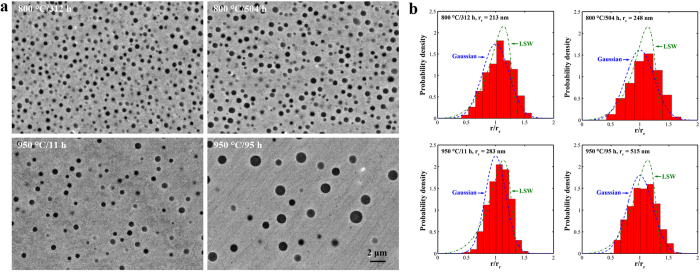
NiAl-type precipitates and their size distribution. (**a**) NiAl-type precipitates characterized by back-scattered electrons in samples aged at 800 and 950 °C. (**b**) Corresponding precipitate-size distribution as in (**a**).

Figure 1(a) demonstrates the morphology evolution of NiAl-type precipitates subjected to aging treatments at 800 and 950 °C. Corresponding precipitate-size distributions are presented in Fig. 1(b). NiAl-type precipitates with a spherical shape are embedded in the matrix, and they remain spherical even at the mean size of ~1.3 μm (950 °C/95 h), which probably results from the small matrix/precipitate lattice mismatch (~0.02% at 700 °C^11^). The results derived from the image analysis are listed in Supplementary Table S1. Note that the mean radius on the observed plane section, *r*_*r*_, was divided by 0.82 and converted to the mean precipitate radius, 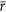
9. At least 534 precipitates were analyzed for each case. Precipitates coarsen during the aging treatment. For example, 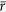 increases from 259 ± 5 nm (800 °C/312 h) to 303 ± 5 nm (800 °C/504 h). Statistical parameters (i.e., variance, skewness, and kurtosis) of the precipitate-size distributions are also included in Supplementary Table S1. The measured size distributions are broader and more symmetrical than the LSW distribution.

### Compositions of the matrix and precipitates

**Figure 2:**
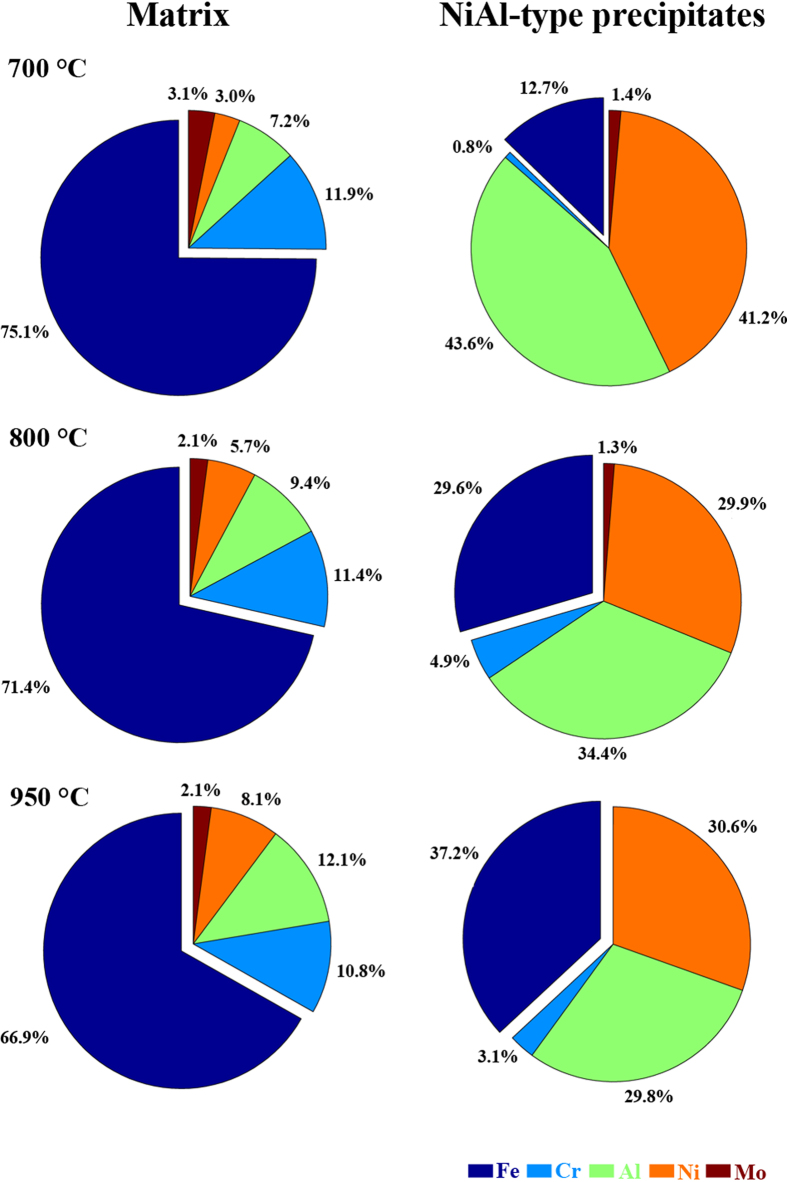
Matrix and precipitate compositions. Compositions (at.%) of the matrix and NiAl-type precipitates for samples aged at 700 °C/100 h, 800 °C/504 h, and 950 °C/95 h. Note that calculated precipitate compositions are presented.

NiAl-type precipitates are gradually dissolved into the matrix, as the aging temperature increases. The precipitate volume fractions (f) are 0.155 ± 0.005^39^, 0.146 ± 0.009, and 0.05 ± 0.005 at 700, 800, and 950 °C, respectively. The solvus temperature of NiAl-type precipitates in FBB8 lies between 950 and 1,000 °C. The compositions of the matrix and precipitates are presented in Fig. 2 and Supplementary Table S2. NiAl-type precipitates are not stoichiometric and display solubility of other elements (i.e., Fe, Cr, and Mo). For example, NiAl-type precipitates have 12.7 ± 0.1 at.% Fe at 700 °C. It is worth noting that the Fe solubility in NiAl-type precipitates at 800 and 950 °C (~30–40 at.%) is approximately three times that at 700 °C. Meanwhile, greater amounts of Cr, but less of Mo, are dissolved in precipitates at higher temperatures. The mole ratio of Al and Ni in NiAl-type precipitates is close to 1:1, suggesting that other solute atoms randomly replace Al and Ni without the clear preferential site occupancy^40^. On the other hand, aging at higher temperatures increases Al and Ni contents in the matrix. For instance, the Al content in the matrix increases from 7.2 ± 0.6 at.% at 700 °C to 12.1 ± 0.2 at.% at 950 °C.

### Evolution of USAXS spectra

**Figure 3:**
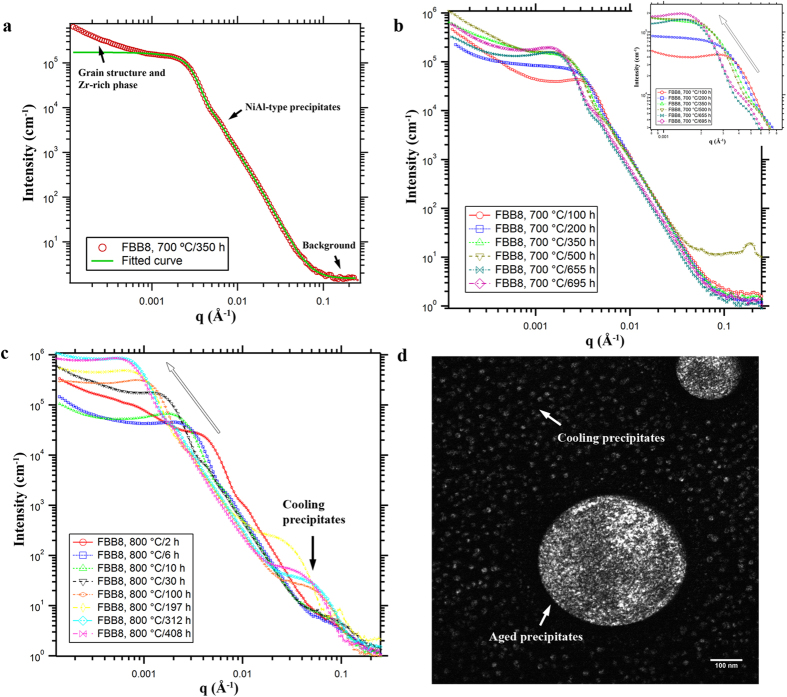
Evolution of USAXS spectra. (**a**) Example of USAXS modeling for the specimen aged for 350 h at 700 °C. (**b**,**c**) Progression of USAXS curves for samples aged at 700 and 800 °C, respectively. (**d**) Duplex NiAl-type precipitates in the sample aged for 100 h at 800 °C + air cooling. The dark-field image was taken using the [100] superlattice reflection.

Figure 3(a) displays a representative USAXS spectrum (700 °C/350 h) as a plot of the scattering intensity, I, vs. the scattering vector, q, on the log-log scale. The spectrum in Fig. 3(a) consists of three regions. In q < 0.001 Å^−1^, the intensity is believed to be dominated by that from the grain structure and Zr-rich minor phase^32^. The main features of NiAl-type precipitates (the Guinier knee and subsequent power slope) are presented in the q range from 0.001 Å^−1^ to 0.07 Å^−1^. In q > 0.07 Å^−1^, the background scattering overwhelms the overall intensity. For this case, the value of 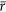 is determined to be ~72 ± 5 nm based on data modeling.

The progression of USAXS spectra as a function of aging time at 700 and 800 °C is presented in Fig. 3(b,c), respectively. Since q is inversely related to the real-space dimension^41^, large precipitates are expected to have scattering features in a small q region. As shown in Fig. 3(b,c), features from aged NiAl-type precipitates shift to the small q region as the aging time increases, indicating that the precipitate size increases during aging treatments. The USAXS-derived results at 700 and 800 °C are listed in Supplementary Tables S3 and S4, respectively. For comparison, we include the ideal inter-precipitate distance, *L*^*ideal*^, defined as


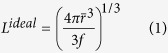


As shown in Supplementary Tables S3 and S4, the inter-precipitate distance of NiAl-type precipitates, L, increases with aging time. For example, L increases from 119 ± 3 nm (700 °C/100 h) to 230 ± 5 nm (700 °C/695 h). The values of L are smaller than those of *L*^*ideal*^, suggesting the existence of a non-uniform spatial distribution of NiAl-type precipitates^42^.

In Fig. 3(c), the scattering curves show additional features in the q range from ~0.01 to 0.1 Å^−1^. These features originate from ultra-fine precipitates with a size ranging from several to tens of nanometers. These ultra-fine precipitates are known to nucleate and grow during the cooling process following the aging treatment and their size depends on the cooling rate^43^. Figure 3(d) displays the dark-field TEM image of the specimen aged for 100 h at 800 °C, followed by air cooling. It reveals duplex precipitates: aged (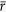 ~ 163 nm) and fine cooling ones (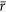 ~ 7.6 nm). Effects of fine cooling precipitates on the room-temperature deformation of NiAl-strengthened ferritic alloys were reported by Sun *et al.*^43^.

## Discussion

**Figure 4:**
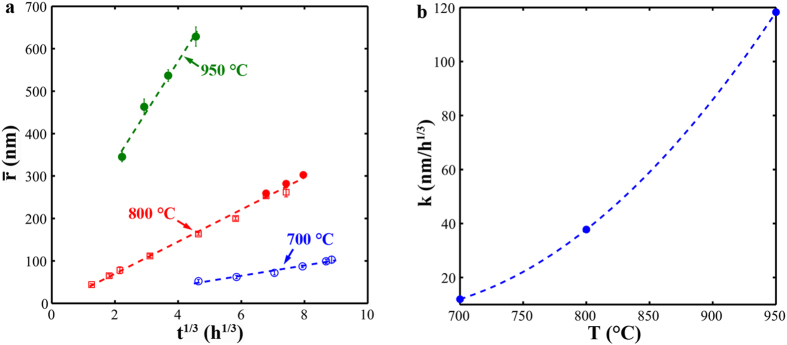
Coarsening kinetics of NiAl-type precipitates. (**a**) Evolution of 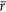 as a function of *t*^1/3^ for three temperatures (open: USAXS; filled: image analysis). (**b**) Coarsening rate, k, as a function of aging temperatures.

The coarsening kinetics in multicomponent alloys can be described by^26^


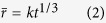


where k is the coarsening rate, and t is the aging time. In Fig. 4(a), 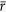 is plotted against *t*^1/3^ for three aging temperatures. The linear relationship between 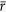 and *t*^1/3^ is well satisfied following Eq. (2). k is determined from the linear slope in Fig. 4(a), giving values of ~12.1, 37.8, and 118.3 nm/h^1/3^ at 700, 800, and 950 °C, respectively [Fig. 4(b)]. The values of 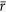 at 800 °C determined by USAXS agree with those derived from image analysis, which supports the USAXS analysis. The consistency between the experimental results and theoretical predictions probably results from the absence of large coherency strain in the matrix^30^. Following the coarsening rate, the precipitate size would be ~1 μm after a ten-year service at 700 °C. Correspondingly, the Orowan stress, Δ*σ*_*Or*_, provided by NiAl-type precipitates would decrease from ~500 MPa (700 °C/100 h) to ~70 MPa (700 °C/10 years)^43^,^44^.

Neglecting off-diagonal terms of the mobility matrix (M), k^3^ is given by^26^


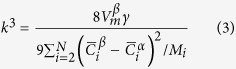


where 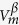 is the precipitate molar volume, *γ* is the matrix/precipitate interfacial energy, M_i_ is the mobility of the component i, and 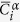 and 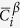 are the equilibrium mole fractions of the component i in the matrix (subscript ‘α’) and NiAl-type precipitates (subscript ‘β’), respectively. The relationship between the mobility matrix, M, and diffusion matrix, D, can be expressed as (neglecting off-diagonal terms)^26^


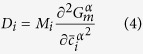


where D_i_ is the self-diffusion or impurity diffusion coefficient of the element i in the matrix and 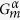 is the matrix molar Gibbs free energy. Assuming the ideal solution for simplicity, 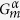 is given by


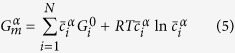


where 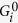 is the molar Gibbs free energy of a pure component i, R is the gas constant, and T is the temperature. Note that 
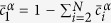
. Hence, the number of component variables is N-1. In this case, 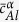, 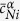, 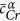, and 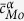 are taken as component variables. The second differential term in Eq. (4) is approximated by


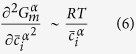


Therefore, Eq. (3) can be expressed as


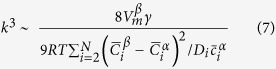


For a ternary alloy system, Eq. (7) is reduced to that developed by Kuehmann and Voorhees^23^. According to Eq. (7), *γ* can be estimated, given other parameters listed in Table 2. 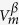 was calculated from the precipitate lattice constant measured by neutron diffraction^11^. Impurity diffusion coefficients in α-Fe were summarized by Abe *et al.*^45^ and original references^46^,^47^,^48^,^49^,^50^ are given in Table 2. By substituting all parameters into Eq. (7), the *γ* values are determined to be ~190, 40, and 20 mJ/m^2^ at 700, 800, and 950 °C, respectively. It is interesting to notice that *γ* at 700 °C is almost at the upper limit of interfacial energy for coherent interface (~200 mJ/m^2^)^51^. Ghosh^52^ calculated *γ* between α-Fe and NiAl by first principles and the computational results (~100, 120, and 250 mJ/m^2^ for {110}, {111}, and {100} habits, respectively) are comparable with the present value at 700 °C.

The interfacial energy between two phases consists of two terms: chemical (*γ*_*ch*_) and structural (*γ*_*st*_) contributions. *γ*_*ch*_ is believed to dominate *γ* due to the small matrix/precipitate lattice mismatch in FBB8^36^. We define the composition variance between the matrix and precipitates (Δ) as


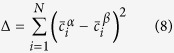


The Δ values at 700, 800, and 950 °C are calculated to be ~0.68, 0.30, and 0.18, respectively. It is concluded that the matrix/precipitate composition difference gradually diminishes as the aging temperature increases. Consequently, the average chemical bonding strength of the matrix and NiAl-type precipitates becomes similar with an increasing aging temperature, which is likely to cause the observed *γ* gap from 700 to 950 °C. Calderon and Fine^36^ investigated the coarsening kinetics of NiAl-type precipitates in Fe-3.00Ni-9.67Al (at.%) at 700 °C and determined k (~5.3 nm/h^1/3^) and *γ* (~20 mJ/m^2^) between the matrix and NiAl-type precipitates. These values are much smaller than those in the present study. Based on the Fe-Ni-Al ternary phase diagram^53^,^54^, NiAl-type precipitates in Fe-3.00Ni-9.67Al (at.%) contains ~30 at.% Fe at 700 °C, which is probably why Calderon and Fine^36^ had a much smaller *γ* and hence, the slower k (Eq. 7).

Based on Eq. (7), precipitate stability in multicomponent alloys is controlled by three factors: the interfacial energy, *γ*, element diffusivities in the matrix, *D*_*i*_, and element partitioning between the matrix and precipitates, 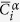 and 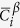. In the development of NiAl-strengthened ferritic alloys, Mo was added to adjust the matrix/precipitate lattice mismatch^38^ to minimize *γ*. However, Calderon *et al.*^36^,^37^ claimed that coherency strain, which results from the matrix/precipitate lattice mismatch (0.06%–0.8% in their studies), has no significant influence on γ, as long as the interface remains coherent. Further, Krug and Dunand^55^ concluded that the interaction between dislocations and precipitates during dislocation climbing in creep is proportional to the matrix/precipitate lattice mismatch. Therefore, it would be effective to control the composition difference (probably by adjusting the Al/Ni ratio) rather than lattice mismatch between the matrix and precipitates in order to minimize *γ*, and hence, k, in NiAl-strengthened ferritic alloys. On the other hand, elements, which have low diffusivities in the matrix and are preferred for enriching precipitates, would help stabilize precipitates. As an interesting example, Wang *et al.*^56^ and Miller *et al.*^57^ reported the unusual stability of Y-Ti-O nanoclusters up to 1,300 °C in ferritic alloys prepared by mechanical alloying. They attributed the observed stability to low diffusivities and solubility of solute atoms due to the strong chemical bonding between solute atoms and vacancies^56^,^58^. Computational calculations^17^,^59^,^60^ would be helpful in order to find such alloying elements that could further retard the coarsening kinetics of NiAl-type precipitates in bcc-iron. For example, Jiao *et al.*^17^ studied the Mn partitioning between the matrix and precipitates in NiAl-strengthened ferritic steels by combined experimental and computational methods. Ding *et al.*^59^ examined the impurity diffusivities of 5d transition metal solutes (Ta - Au) in α-Fe by first-principles calculations and found that they have higher or similar diffusivities in α-Fe, as compared to the Fe self-diffusivity, except Re and Os.

The thermal instability of nano-sized strengthening features in high-temperature materials (e.g., nickel-based superalloys and advanced heat-resistant steels) significantly shortens materials’ service life at elevated temperatures. By illustrating the controlling factors in the ripening process of multicomponent alloys, the present study provides insights into designing and developing advanced materials with stable microstructures for long-term service at high temperatures.

In conclusion, we have studied the stability of nano-sized NiAl-type precipitates in FBB8 at 700–950 °C by USAXS and electron microscopies. The linear relationship between 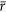 and *t*^1/3^ is well satisfied, which is consistent with the ripening model in multicomponent alloys^26^. The coarsening rates were determined to be ~12.1, 37.8, and 118.3 nm/h^1/3^ at 700, 800, and 950 °C, respectively. Correspondingly, the interfacial energies were estimated to be ~190, 40, and 20 mJ/m^2^ at three aging temperatures. We found that interfacial energy is dominated by the composition difference rather than lattice mismatch between the matrix and precipitates in NiAl-strengthened ferritic alloys. This study profiles the ripening processing in multicomponent alloys by discussing the controlling factors of interfacial energy, diffusivities, and element partitioning, which provides guidelines to design and develop advanced materials with stable microstructures.

## Methods

### Materials preparation

An ingot of FBB8 (~12.7 cm × 25.4 cm × 1.9 cm) was prepared by Sophisticated Alloys, Inc. using vacuum-induction melting. Hot iso-thermal pressing (HIP) at 1,200 °C/103 MPa for 4 h was performed to minimize casting porosity. Slices were cut from the ingot, and sealed into quartz tubes in a vacuum environment. Encapsulated specimens were solution treated for 1 h at 1,200 °C, followed by air cooling, and then aged at 700–950 °C.

### USAXS experiments

USAXS measurements on thin foils (thickness of ~70 μm) were performed with the photon energy of 16.8 keV and slit of 1.5 mm × 0.6 mm at the ChemMatCARS beamline 15-ID-D, located at the Advanced Photon Source (APS), Argonne National Laboratory. The beamline setup was reported elsewhere^61^. Raw data was reduced, using the Indra package, which resulted in data on the absolute intensity scale. The reduced USAXS data was analyzed by Irena^62^. The form factor of spherical particles was used, while the inter-particle structure factor^42^,^63^,^64^ was employed to characterize the interaction among NiAl-type precipitates. The precipitate-size distribution becomes more symmetrical and broader with an increasing volume fraction than does the LSW distribution^65^. Hence, it was assumed that the precipitate-size distribution follows the Gaussian distribution. Result uncertainty was obtained by evaluating the effect of input data uncertainties and stability of fitting parameters.

### Characterization by electron microscopies

Specimens were examined in a Zeiss Gemini 1525 SEM equipped with a back-scattered electron detector. Specimens were polished without chemical etching. Images were converted to binary ones through the image-processing toolbox in Matlab^66^. The information, such as precipitate volume fraction and size distribution, was extracted from binary images. Meanwhile, the compositions of the matrix and precipitates were determined by the energy-dispersive X-ray spectroscopy (EDS) in SEM. At least three measurements were performed in each case. Due to the limited resolution, the EDS measurements were conducted on precipitates with a size larger than ~1 μm. Otherwise, the lever rule^39^ was used to calculate the precipitate compositions, given the precipitate volume fraction.

Moreover, TEM was conducted using a Zeiss Libra 200 model. Discs with the diameter of 3 mm were polished to ~70 μm, and then further thinned through electro-polishing in a Fischione twin-jet polisher. The electrolyte of 5 volume percent HCl in ethanol was used at room temperature. After perforation, the TEM specimens were ion milled for ~15 minutes with the ion energy of 3.5 kV.

## Additional Information

**How to cite this article**: Sun, Z. *et al.* Nano-sized precipitate stability and its controlling factors in a NiAl-strengthened ferritic alloy. *Sci. Rep.* **5**, 16081; doi: 10.1038/srep16081 (2015).

## Supplementary Material

Supplementary Information

## Figures and Tables

**Table 1:** The compositions of the FBB8 ingot, atomic percent (at.%).

	Al	Ni	Cr	Mo	Zr	B	Fe
Nominal Composition	12.72	9.00	10.15	1.87	0.2	0.02	Bal.
Measured Composition	13.02	9.25	10.45	1.96	0.2	0.02	Bal.

**Table 2:** List of parameters and estimated interfacial energies at 700, 800, and 950 °C.

	700 °C	800 °C	950 °C
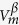 (m^3^/mol)	1.47 × 10^−5^	1.48 × 10^−5^	1.49 × 10^−5^
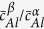	0.436/0.072^39^	0.344/0.094	0.303/0.121
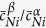	0.412/0.03^39^	0.299/0.057	0.299/0.081
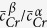	0.008/0.119^39^	0.049/0.114	0.049/0.108
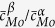	0.014/0.031^39^	0.013/0.021	0.007/0.021
D_Al_ (m^2^ s^−1^)	3.6 × 10^−17 ^ 46	5.5 × 10^−16 ^ 47	1.6 × 10^−14 ^ 47
D_Ni_ (m^2^ s^−1^)^48^	8.9 × 10^−18^	3.8 × 10^−16^	1.5 × 10^−14^
D_Cr_ (m^2^ s^−1^)^49^	4.0 × 10^−18^	3.7 × 10^−16^	1.5 × 10^−14^
D_Mo_ (m^2^ s^−1^)^50^	4.1 × 10^−18^	2.5 × 10^−16^	1.2 × 10^−14^
*γ* (mJ/m^2^)	~190	~40	~20

## References

[b1] Viswanathan R. & Bakker W. Materials for ultrasupercritical coal power plants - boiler materials: Part 1. J. Mater. Eng. Perform. 10, 81–95 (2001).

[b2] Viswanathan R. & Bakker W. Materials for ultrasupercritical coal power plants - turbine materials: Part II. J. Mater. Eng. Perform. 10, 96–101 (2001).

[b3] Viswanathan R. *et al.* U. S. program on materials technology for ultra-supercritical coal power plants. J. Mater. Eng. Perform. 14, 281–292 (2005).

[b4] Bhadeshia H. K. D. H. Design of ferritic creep-resistant steel. ISIJ Int. 41, 626–640 (2001).

[b5] Von Hagen I. & Bendick W. Creep resistant ferritic steels for power plants. In International Symposium on Niobium 2001, 753–776 (2001).

[b6] Taneike M., Abe F. & Sawada K. Creep-strengthening of steel at high temperatures using nano-sized carbonitride dispersions. Nature 424, 294–296 (2003). 12867976 10.1038/nature01740

[b7] Ardell A. J. Precipitation hardening. Metall. Trans. A 16, 2131–2165 (1985).

[b8] Gleiter H. & Hornbogen E. Precipitation hardening by coherent particles. Mater. Sci. Eng. 2, 285–302 (1967/68).

[b9] Nembach E. Particle Strengthening of Metals and Alloys. (John Wiley & Sons, Inc., 1997).

[b10] Hirata A. *et al.* Atomic structure of nanoclusters in oxide-dispersion-strengthened steels. Nature Mater. 10, 922–926 (2011). 22019943 10.1038/nmat3150

[b11] Huang S. *et al.* *In situ* neutron-diffraction studies on the creep behavior of a ferritic superalloy. Metall. Trans. A 43, 1497–1508 (2011).

[b12] Huang S. *et al.* Deformation mechanisms in a precipitation-strengthened ferritic superalloy revealed by *in situ* neutron diffraction studies at elevated temperatures. Acta Mater. 83, 137–148 (2015).

[b13] Stallybrass C., Schneider A. & Sauthoff G. The strengthening effect of (Ni,Fe)Al precipitates on the mechanical properties at high temperatures of ferritic Fe–Al–Ni–Cr alloys. Intermetallics 13, 1263–1268 (2005).

[b14] Teng Z. K. *et al.* New NiAl-strengthened ferritic steels with balanced creep resistance and ductility designed by coupling thermodynamic calculations with focused experiments. Intermetallics 29, 110–115 (2012).

[b15] Vo N. Q., Liebscher C. H., Rawlings M. J. S., Asta M. & Dunand D. C. Creep properties and microstructure of a precipitation-strengthened ferritic Fe–Al–Ni–Cr alloy. Acta Mater. 71, 89–99 (2014).

[b16] Zhu S. M., Tjong S. C. & Lai J. K. L. Creep behavior of a NiAl precipitation strengthened ferritic Fe-Cr-Ni-Al alloy. Acta Mater. 46, 2969–2976 (1998).

[b17] Jiao Z. B., Luan J. H., Miller M. K., Yu C. Y. & Liu C. T. Effects of Mn partitioning on nanoscale precipitation and mechanical properties of ferritic steels strengthened by NiAl nanoparticles. Acta Mater. 84, 283–291 (2015).

[b18] Taillard R. & Pineau A. The precipitation of the intermetallic compound NiAl in Fe-19 wt.%Cr allloys. Mater. Sci. Eng. 54, 209–219 (1982).

[b19] Ratke L. & Voorhees P. W. Growth and Coarsening: Ostwald Ripening in Material Processing. (Springer Science & Business Media, 2002).

[b20] Lifshitz I. M. & Slyozov V. V. The kinetics of precipitation from supersaturated solid solutions. J. Phys. Chem. Solids 19, 35–50 (1961).

[b21] Wagner C. Theory of precipitate change by redissolution. Z. Elektrochem. 65, 581–591 (1961).

[b22] Bjorklund S., Donaghey L. F. & Hillert M. The effect of alloying elements on the rate of Ostwald ripening of cementite in steel. Acta Metall. 20, 867–874 (1972).

[b23] Kuehmann C. J. & Voorhees P. W. Ostwald ripeing in ternary alloys. Metall. Trans. A 27, 937–943 (1996).

[b24] Morral J. E. & Purdy G. R. Particle coarsening in binary and multicomponent alloys. Scripta Metall. 30, 905–908 (1994).

[b25] Umantsev A. & Olson G. B. Ostwald ripening in multicomponent alloys. Scripta Metall. 29, 1135–1140 (1993).

[b26] Philippe T. & Voorhees P. W. Ostwald ripening in multicomponent alloys. Acta Mater. 61, 4237–4244 (2013).

[b27] Maruyama K., Sawada K. & KoiKe J.-I. Strengthening mechanisms of creep resistant tempered martensitic steel. ISIJ Int. 41, 641–653 (2001).

[b28] Pešička J., Kužel R., Dronhofer A. & Eggeler G. The evolution of dislocation density during heat treatment and creep of tempered martensite ferritic steels. Acta Mater. 51, 4847–4862 (2003).

[b29] Sawada K. *et al.* Effect of W on recovery of lath strucutre during creep of high chromium martensitic steels. Mater. Sci. Eng. A 267, 19–25 (1999).

[b30] Ardell A. J. & Nicholson R. B. The coarsening of r′ in Ni-Al alloys. J. Phys. Chem. Solids 27, 1793–1804 (1966).

[b31] Li X., Saunders N. & Miodownik A. P. The coarsening kinetic of r’ particles in nickel-based alloys. Metall. Trans. A 33, 3367–3373 (2002).

[b32] Kelekanjeri V. S. K. G., Moss L. K., Gerhardt R. A. & Ilavsky J. Quantification of the coarsening kinetics of r’ precipitates in Waspaloy microstructures with different prior homogenizing treatments. Acta Mater. 57, 4658–4670 (2009).

[b33] Abe F. & Nakazawa S. The effect of tungsten on creep behavior of tempered martensitic 9Cr Steels. Metall. Trans. A 23, 3025–3034 (1992).

[b34] Yoshizawa M. *et al.* Effect of precipitates on long-term creep deformation properties of P92 and P122 type advanced ferritic steels for USC power plants. Mater. Sci. Eng. A 510-511, 162–168 (2009).

[b35] Ennis P. J. & Czyrska-Filemonowicz A. Recent advances in creep-resistant steels for power plant applications. Sadhana 28, 709–730 (2003).

[b36] Calderon H. & Fine M. E. Coarsening kinetics of coherent NiAl-type precipitates in Fe-Ni-Al and Fe-Ni-Al-Mo alloys. Mater. Sci. Eng. 63, 197–208 (1984).

[b37] Calderon H., Fine M. E. & Weertman J. R. in *7th International Conference on the Strength of Metals and Alloys.* (eds H. J. McQueen, J.-P. Bailon & J. I. Dickson) 737-742 (Pergamon, Oxford).

[b38] Calderon H., Fine M. E. & Weertman J. R. Coarsening and morphology of NiAl particles in Fe-Ni-Al-Mo ferritic alloys. Metall. Trans. A 19, 1135–1146 (1988).

[b39] Teng Z. K. *et al.* Characterization of nanoscale NiAl-type precipitates in a ferritic steel by electron microscopy and atom probe tomography. Scripta Mater. 63, 61–64 (2010).

[b40] Anderson I. M., Duncan A. J. & Bentley J. Site-distributions of Fe alloying additions to B2-ordered NiAl. Intermetallics 7, 1017–1024 (1999).

[b41] Guinier A. & Fournet G. Small-Angle Scattering of X-ray. (Wiley, 1955).

[b42] Giordano R., Grasso A., Teixeira J., Wanderlingh F. & Wanderlingh U. Small-angle neutron scattering in lysozyme solutions. Phys. Rev. A 43, 6894–6899 (1991). 9905039 10.1103/physreva.43.6894

[b43] Sun Z., Song G., Ilavsky J. & Liaw P. K. Duplex precipitates and their effects on the room-temperature fracture behaviour of a NiAl-strengthened ferritic alloy. Mater. Res. Lett. 3, 128–134 (2015).

[b44] Martin J. W. Micromechanisms in Particle-Hardened Alloys. (Cambridge University Press, 1980).

[b45] Abe F., Kern T.-U. & Viswanathan R. Creep-Resistant Steels. (Woodhead Publishing 2008).

[b46] Philbert J. La Diffusion dans les Solides (Presses Universitaires de France, 1966).

[b47] Nishida K., Yamamoto T. & Nagata T. On the interdiffusion in α-solid solution of the Fe-Al system in Al vapor. Trans. JIM 12, 310–316 (1971).

[b48] Cermak J., Lubbehusen M. & Mehrer H. The influence of the magnetic phase transformation on the heterodiffusion of exp 63 Ni in alpha-Iron. Z. Metallkd. 80, 213–219 (1989).

[b49] Lee C. G., Iijima Y., Hiratani T. & Hirano K. Diffusion of chromium in α-iron. Mater. Trans., JIM 31, 255–261 (1990).

[b50] Nitta H. *et al.* Diffusion of molybdenum in α-iron. Acta Mater. 50, 4117–4125 (2002).

[b51] Porter D. A. & Easterling K. E. Phase transformations in metals and alloys. Second edn, 315–317 (Chapman & Hall, 1992).

[b52] Liaw P. K. *et al.* *Computational and experimental design of Fe-based superalloys for elevated-temperature applications. Technical report*. (2012) Available at: http://www.osti.gov/scitech/biblio/1047697. (Accessed: 13th September 2015).

[b53] Bradley A. J. & Taylor A. An X-ray study of the iron-nickel-aluminium ternary equilibrium diagram. Proc. Roy. Soc. A 166, 353–375 (1938).

[b54] Bradley A. J. Microscopical studies on the iron-nickel-aluminium system. J. Iron Steel Inst. 168, 233–244 (1951).

[b55] Krug M. E. & Dunand D. C. Modeling the creep threshold stress due to climb of a dislocation in the stress field of a misfitting precipitate. Acta Mater. 59, 5125–5134 (2011).

[b56] Wang X. L. *et al.* Unusual thermal stability of nano-structured ferritic alloys. J. Alloys Compd. 529, 96–101 (2012).

[b57] Miller M. K., Hoelzer D. T., Kenik E. A. & Russell K. F. Stability of ferritic MA/ODS alloys at high temperatures. Intermetallics 13, 387–392 (2005).

[b58] Fu C. L., Krčmar M., Painter G. S. & Chen X.-Q. Vacancy mechanism of high oxygen solubility and nucleation of stable oxygen-enriched clusters in Fe. Phys. Rev. Lett. 99 (2007). 10.1103/PhysRevLett.99.225502 18233295

[b59] Ding H., Huang S., Ghosh G., Liaw P. K. & Asta M. A computational study of impurity diffusivities for 5d transition metal solutes in α-Fe. Scripta Mater. 67, 732–735 (2012).

[b60] Huang S. *et al.* Calculation of impurity diffusivities in α-Fe using first-principles methods. Acta Mater. 58, 1982–1993 (2010).

[b61] Ilavsky J. *et al.* Ultra-small-angle X-ray scattering at the Advanced Photon Source. J. Appl. Crystallogr. 42, 469–479 (2009).

[b62] Ilavsky J. & Jemian P. R. Irena: tool suite for modeling and analysis of small-angle scattering. J. Appl. Crystallogr. 42, 347–353 (2009).

[b63] Farsaci F. *et al.* Dynamical behaviour of structured macromolecular solutions. Phys. Chem. Liq. 20, 205–220 (1989).

[b64] Huang E. W. *et al.* Study of nanoprecipitates in a nickel-based superalloy using small-angle neutron scattering and transmission electron microscopy. Appl. Phys. Lett. 93, 161904 (2008).

[b65] Akaiwa N. & Voorhees P. W. Late-stage phase separation: dynamics, spatial correlations, and structure functions. Phys. Rev. E 49, 3860–3880 (1994). 10.1103/physreve.49.3860 9961674

[b66] The MathWorks, Inc. Available at: http://www.mathworks.com. (Accessed: 13th September 2015).

