# Supplementary material for: Nano-sized precipitate stability and its controlling factors in a NiAl-strengthened ferritic alloy
Source: Sci Rep. 2015 Nov 5;5:16081. doi: 10.1038/srep16081 (PMC4633603; doi:10.1038/srep16081)
Supplement: Supplementary Information [file srep16081-s1.pdf]

Supplementary information for

Nano-sized precipitate stability and its controlling factors in a NiAl-strengthened ferritic alloy

Zhiqian Sun[1], Gian Song[1], Jan Ilavsky[2], Gautam Ghosh[3], and Peter K. Liaw[1,*]

[1] Department of Materials Science and Engineering, The University of Tennessee, Knoxville, TN 37996, USA

[2] X-ray Operations Division, Argonne National Laboratory, Lemont, IL 60439, USA

[3] Department of Materials Science and Engineering, Northwestern University, Evanston, IL 60208, USA

*Corresponding author. Email: pliaw@utk.edu

Table S1. Results from image analysis (T: aging temperature; t: time; N: population of accounted precipitates; r_r: mean precipitate radius on the observed plane section; r_{max}/r_{min}: maximum/minimum radii on the observed plane section; std: standard deviation of the precipitate radius on the observed plane section; $\bar{r}$: mean precipitate radius).

T (℃)	t (h)	N	r_r (nm)	r_{max}/r_{min} (nm)	std (nm)	$\bar{r}$ (nm)	Variance	Skewness	Kurtosis
800	312	534	213 ± 4	331/75	49	259 ± 5	0.053	- 0.18	2.56
800	408	728	231 ± 4	356/36	52	282 ± 4	0.051	- 0.33	2.90
800	504	960	248 ± 4	445/44	61	303 ± 5	0.061	- 0.16	2.61
950	11	853	283 ± 9	426/112	50	345 ± 11	0.032	- 0.21	2.71
950	25	690	380 ± 15	556/107	83	463 ± 19	0.047	- 0.36	2.90
950	50	1270	440 ± 11	678/230	78	537 ± 14	0.032	0.014	2.38
950	95	1164	515 ± 19	844/254	114	629 ± 23	0.049	0.030	2.33
LSW distribution							0.046	- 0.92	3.67

Table S2. Compositions of the bcc iron matrix and NiAl-type precipitates, at.%. The values in brackets are calculated by the lever rule. The data at 700 ℃ was reported by Teng et al. (Scripta Mater. 63, 61-64, 2010).

		Fe	Cr	Al	Ni	Mo
700 ℃	precipitates	12.7 ± 0.1	0.8 ± 0.1	43.6 ± 0.9	41.2 ± 1.1	1.4 ± 0.1
	matrix	75.1 ± 1.0	11.9 ± 0.7	7.2 ± 0.6	3.0 ± 0.5	3.1 ± 0.4
800 ℃	precipitates	(29.6 ± 4.8)	(4.9 ± 0.7)	(34.4 ± 1.6)	(29.9 ± 3.8)	(1.3 ± 1.8)
	matrix	71.4 ± 0.7	11.4 ± 0.1	9.4 ± 0.1	5.7 ± 0.6	2.1 ± 0.3
950 ℃	precipitates	34.3 ± 2.5<br>(37.2 ± 5.6)	4.9 ± 0.7<br>(3.1 ± 4.0)	30.3 ± 0.6<br>(29.8 ± 3.4)	29.9 ± 2.7<br>(30.6 ± 5.0)	0.7 ± 0.1<br>-
	matrix	66.9 ± 0.3	10.8 ± 0.2	12.1 ± 0.2	8.1 ± 0.2	2.1 ± 0.1

Table S3. USAXS-derived parameters for specimens aged at 700 ℃ (std: standard deviation of $\bar{r}$: L: inter-precipitate distance; and σ: standard deviation of L).

t (h)	$\bar{r}$ (nm)	std (nm)	L (nm)	σ (nm)	L^{ideal} (nm)
100	52 $\pm$ 4	13 $\pm$ 2	119 $\pm$ 3	64 $\pm$ 2	156
200	62 $\pm$ 4	20 $\pm$ 2	131 $\pm$ 4	80 $\pm$ 1	186
350	72 $\pm$ 5	21 $\pm$ 3	150 $\pm$ 8	115 $\pm$ 2	216
500	87 $\pm$ 3	19 $\pm$ 1	156 $\pm$ 4	107 $\pm$ 1	261
655	99 $\pm$ 5	22 $\pm$ 3	235 $\pm$ 6	122 $\pm$ 3	297
695	103 $\pm$ 8	28 $\pm$ 4	230 $\pm$ 5	119 $\pm$ 2	309

Table S4. USAXS-derived parameters for specimens aged at 800 ℃ (std: standard deviation of $\bar{r}$: L: inter-precipitate distance; and σ: standard deviation of L).

t (h)	$\bar{r}$ (nm)	std (nm)	L (nm)	σ (nm)	L^{ideal} (nm)
2	44 ± 2	10 ± 1	90 ± 11	69 ± 4	135
6	65 ± 4	14 ± 2	130 ± 18	95 ± 7	199
10	78 ± 8	16 ± 2	140 ± 29	116 ± 10	239
30	112 ± 4	24 ± 2	220 ± 17	159 ± 5	343
100	163 ± 3	31 ± 3	340 ± 17	240 ± 12	499
197	200 ± 4	42 ± 2	360 ± 23	258 ± 8	612
312	253 ± 4	39 ± 1	370 ± 20	253 ± 4	775
408	262 ± 11	70 ± 11	460 ± 44	290 ± 14	802
